# A Validation Employing Convolutional Neural Network for the Radiographic Detection of Absence or Presence of Teeth

**DOI:** 10.3390/jcm10061186

**Published:** 2021-03-12

**Authors:** María Prados-Privado, Javier García Villalón, Antonio Blázquez Torres, Carlos Hugo Martínez-Martínez, Carlos Ivorra

**Affiliations:** 1 Asisa Dental, Research Department, C/José Abascal, 32, 28003 Madrid, Spain; javier.villalon@asisadental.com (J.G.V.); antonio.blazquez@colaboradorasisadental.com (A.B.T.); carlos.martinez@asisa.es (C.H.M.-M.); carlos.ivorra@asisadental.com (C.I.); 2 Department of Signal Theory and Communications, Higher Polytechnic School, Universidad de Alcalá de Henares, Ctra. Madrid-Barcelona, Km. 33,600, 28805 Alcala de Henares, Spain; 3 Department Continuum Mechanics and Structural Analysis, Higher Polytechnic School, Carlos III University, Avenida de la Universidad 30, Leganés, 28911 Madrid, Spain; 4 SysOnline, 30001 Murcia, Spain; 5 Faculty of Medicine, Universidad Complutense de Madrid, Plaza de Ramón y Cajal, s/n, 28040 Madrid, Spain

**Keywords:** teeth detection, neural network, panoramic images

## Abstract

Dental radiography plays an important role in clinical diagnosis, treatment and making decisions. In recent years, efforts have been made on developing techniques to detect objects in images. The aim of this study was to detect the absence or presence of teeth using an effective convolutional neural network, which reduces calculation times and has success rates greater than 95%. A total of 8000 dental panoramic images were collected. Each image and each tooth was categorized, independently and manually, by two experts with more than three years of experience in general dentistry. The neural network used consists of two main layers: object detection and classification, which is the support of the previous one. A Matterport Mask RCNN was employed in the object detection. A ResNet (Atrous Convolution) was employed in the classification layer. The neural model achieved a total loss of 0.76% (accuracy of 99.24%). The architecture used in the present study returned an almost perfect accuracy in detecting teeth on images from different devices and different pathologies and ages.

## 1. Introduction

A very important element in any field of medicine and, in particular, of dentistry, is to have good clinical diagnosis with which it is possible to make decisions about how the treatment will be executed [1]. An important role in clinical diagnosis, treatment and surgery is played by dental radiography since it can be used to find bone loss, cavities and hidden dental structures among others [2,3]. Sometimes misdiagnosis may occur due to several dentist factors such as fatigue, emotions and low experience levels, but these situations can be reduced thanks to the use of tools based on artificial intelligence to interpret dental X-ray images [4].

Dental images can be classified as intraoral or extraoral images. Panoramic X-ray images are extraoral radiographs that are used to detect dental problems in the jaw and the maxilla. Periapical images or bitewing images are an example of intraoral radiographies with which it is possible to get details of certain area of mouth [2,5].

Panoramic radiographies are very common in dentistry because they allow screening of a broad anatomical region and at the same time require a relatively low radiation dose. However, this kind of image can be sometimes difficult to interpret, especially for inexperienced observers, and therefore, a bad diagnosis can be done [6].

Techniques such as artificial intelligence that provide automated solutions can help professionals in clinical decision-making, in saving time and reducing the effects of fatigue during the daily practice [7]. Convolutional neural networks (CNNs) have been employed with promising results to study cephalometric landmark detection [8] or teeth classification [9].

The different artificial intelligence technologies can be used, and they are been used as a powerful tool to enhance, extend, and expand human capabilities, delivering the types of care patients need, at the time and place they need them [10,11].

If artificial intelligence is employed to help clinicians make decisions, it should be embedded into electronic healthcare records, and thanks to that it would be possible to support evidence-based decision-making to improve the patient experience, and quality and efficiency of the care and communications [10].

Computed tomography (CT) or bitewings are other common images employed in clinical practice and have also been used in artificial intelligence with different objectives [9,12]. However, from the best authors’ knowledge, only Tuzoff et al. employed in their study [3] panoramic radiographies for teeth detection and numbering. In that study, a deep CNN was employed, which is the most popular method applied to image recognition due to its great success in the detection, segmentation and recognition of objects and regions in images [13]. Other authors have employed different segmentation algorithms and image processing to teeth detection [14] such as the contour detection method [15] or level set method [16].

In recent years, deep learning has been developed considerably and now it extracts certain features that are difficult for humans to recognize [4]; currently, deep-learning methods based on CNNs are an important methodology in the field of medical image analysis [17].

The aim of this study was to detect the absence or presence of teeth using an effective convolutional neural network, which reduces calculation times and has success rates greater than 95%.

## 2. Materials and Methods

### 2.1. Study Design

This study used a dataset of anonymized panoramic images. A CNN was constructed to detect the presence or absence of teeth on the radiographies. Reporting of this study follows the STARD guideline [18].

**Figure 1:**
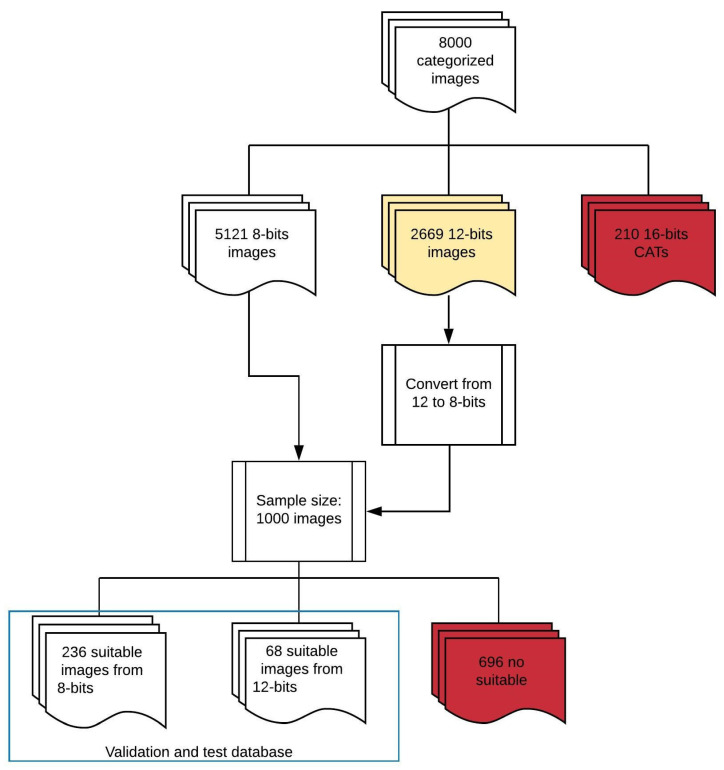
Flowchart followed with the categorized images.

Figure 1 details the work flow of this study. A total of 8000 dental panoramic images were collected and categorized by the experts. A Matterport Mask RCNN and Resnet network were used to train and validate. To finish, images were finally interpreted automatically.

### 2.2. Sample Size Calculation

The sample proposed for this study was chosen to obtain a sufficiently robust database that provides a confidence interval appropriate to the study (greater than 95%) [19]. Based on our hypothesis, a sample size of 8000 images should be analyzed by the examiners.

### 2.3. Image Categorization

**Figure 2:**
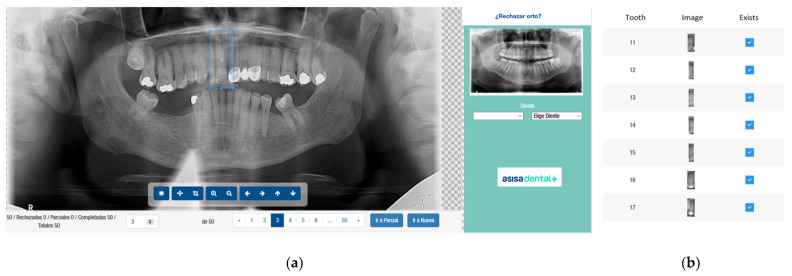
(**a**): Visualization program employed to collect data by the examiners; (**b**) a detail of the visualization program.

For each image and each tooth, two examiners, with more than three years of experience in general dentistry, independently and manually selected the presence or absence of teeth. The presence of a tooth is defined as a crown and root, and the absence of a tooth is defined as no type of crown. The two evaluators analyzed the collection of radiographs through a visualization program created to collect information on each image, as Figure 2 details.

### 2.4. Image Dataset

Panoramic images were taken from Asisa Dental S.A.U. centers in the Community of Madrid (Spain). These images are completely anonymized by CareStream Health Spain SA (Pozuelo de Alarcón, Madrid, Spain). No additional information such as name, gender, age, or when the image was taken appears in the database. Data collection was ethically approved (Ethics Committee of Research with Regional Medicines of the Community of Madrid (CEIm-R)) on 15 June 2018. The requirement to obtain informed consent from patients was waived by the ethics committee.

**Figure 3:**
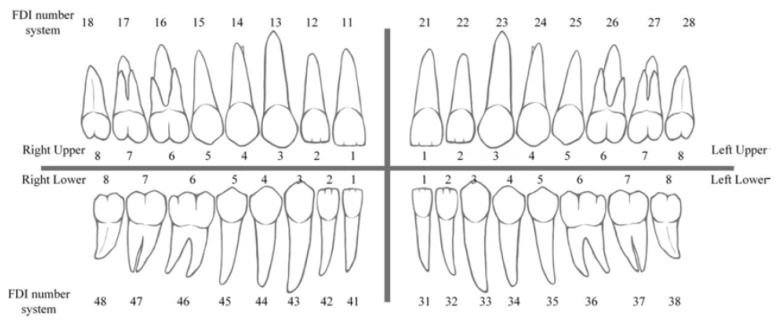
FDI classification.

The radiographies included in the study were those that correspond to adults older than 18. Images of edentulous patients, those with temporary teeth, poor definition (the minimum definition detailed in Table 1), with removable prostheses or with only presence of implants were excluded. Computerized axial tomographies (CAT) were also excluded. Radiographies with overlap or objects out of the imaging plane were excluded. A total of 8000 dental panoramic images were collected and categorized by the experts. The categorization consists of selecting each tooth with an image, indicating the position (“bounding box”) according to FDI classification (Figure 3) and in selecting different variables as explained in Section 2.3.

These 8000 images were divided into training, validation and test datasets once the quality of the categorization was analyzed with the aim of avoiding future settings. Although all images were in DICOM (“*.dcm”) format, some important differences regarding resolution and quality were found. Within the 8000 images of the initial sample, the majority were 8 and 12 bit, with some images of 16 bit that corresponded to CATs, which were excluded from the study. Table 1 details the total image distribution with its resolution.

The database is divided in three groups: train, test and validate. The first two groups are employed to construct the network and the validation is executed with images that the algorithm has never seen. It is necessary first to pass the first group of images through the network to obtain the coefficients of each neuron (dependent and independent) and later it is passed to the validation ones because the algorithm has never seen the data.

**Figure 4:**
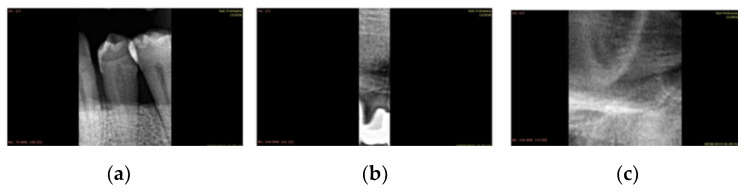
Anomalies in bounding boxes: (**a**) Several teeth in the same bounding box; (**b**) Tooth incorrectly delimited; (**c**) Indistinguishable.

DICOM 12-bit to 8-bit images were homogenized. A sample of 1000 images of 8 bit and 12 bit converted to 8 was selected and the categorization made by the examiners was analyzed one by one. For this, a DICOM image was generated from each panoramic with each tooth according to the coordinates coming from the categorization, and it was checked that they had a minimum quality for the training. Categorized images with anomalies such as those shown in Figure 4 were found. Radiographs with these anomalies were eliminated.

A total of 304 images were employed to train and test the model. The training group was used to train teeth detection, and the testing group was used for evaluation of the performance of the neural network architecture.

### 2.5. CNN Architecture

The categorized panoramic radiographs are used as an input for the neural network architecture presented. The system outputs the bounding boxes for all detected teeth on the image.

The training process was executed on a GPU GTX 1080, with 11 GB memory. The algorithms were running backend on TensorFlow version 1.14, and the operating system was Windows 10 and Ubuntu 18.4. In the final step, it was tested in the cloud (AWS) on instance p3.8large (4 GPU’s Tesla V100, 64 GB GPU memory, instance memory: 244 GB, vCores of the instance: 32), with the Deep Learning AMI using the virtual environment of conda tensorflow_p36.

The neural network used consists of two main layers: object detection and classification, which is the support of the previous one.

**Figure 5:**
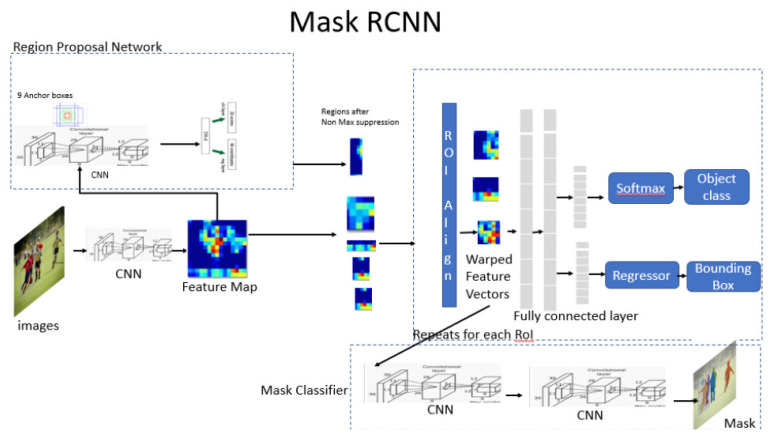
General Mask RCNN architecture.

The main task of object detection is to define the regions of interest where the objects can be located. A Matterport Mask RCNN was employed in the object detection (Figure 5). During the first scan, the neural network proposes regions of analysis. During the second phase, the network classifies the regions and proposes bounding boxes and masks. The object detection process calculates the Region Proposal Network (RPN), the Region of Interest (ROI) and bounding boxes. Finally, ROIs and bounding boxes are improved, and masks are generated.

**Figure 6:**
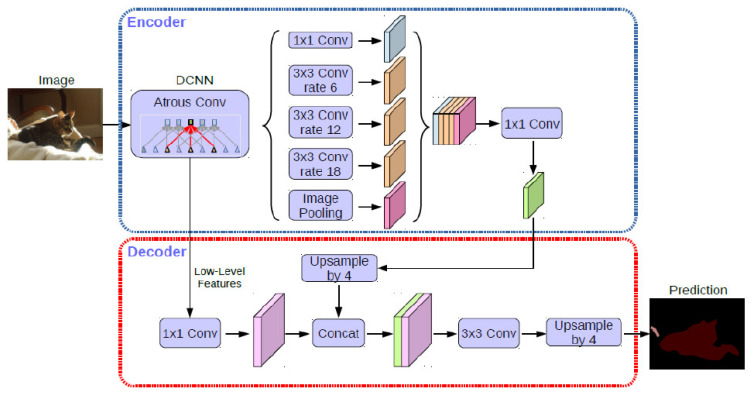
General ResNet Atrous architecture.

A ResNet (Atrous Convolution) was employed in the classification layer (Figure 6).

## 3. Results

### 3.1. Image Categorization Results

The mean age of the 8000 images was 48.93 years with a standard deviation of 17.39 years. Two examiners analyzed independently the same panoramic images with a Cohen Kappa of 0.9 in presence of teeth. Each examiner reanalyzed a total of 50 images. The intraexaminer concordance was *k* = 0.9065 in one of the examiners and *k* = 0.8637 in the other.

### 3.2. Teeth Detection Results

The neural model achieved a total loss of 0.76% (accuracy of 99.24%). This result was obtained with the parameters detailed in Table 2.

**Figure 7:**
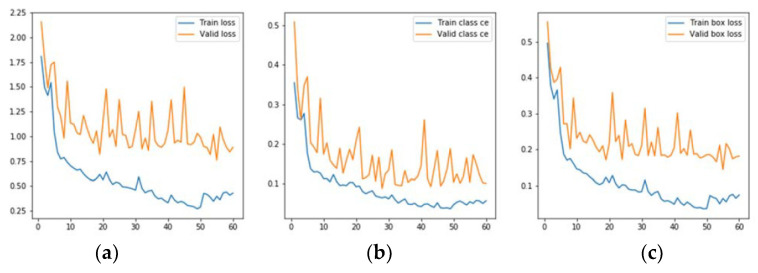
(**a**) total loss; (**b**) class and (**c**) BBox loss of the model.

The evolution graphs of the selected metrics, both of the training set and the validation set, are shown in Figure 7. As can be seen in the deviation of the validation curves over the training ones in Figure 7, there is no overtraining. The blue line represents training data’s behavior and the orange line represents validation data’s behavior. The final value in total loss figure (Figure 7a) was 0.75 in testing and 0.39 in training. The final value in class loss (Figure 7b) was 0.002 in training and 0.006 in testing. Finally, Figure 7c represents a value in BBox loss of 0.064 in training and 0.19 in testing.

### 3.3. Some Interesting Examples

**Figure 8:**
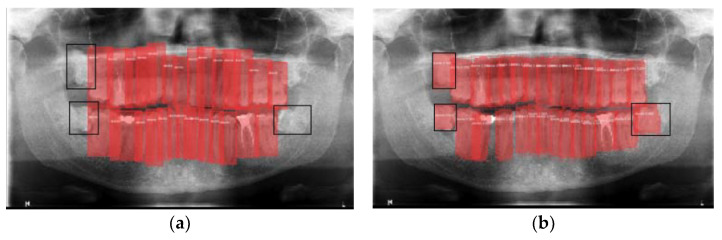
False positives: (**a**) Image categorization; (**b**) Bounding boxes detected by the model.

Figure 8 shows false-positives, which come from the model detecting bounding boxes that do not appear in the categorization of the validation image in question. In this case, the model produces false positives in the form of detecting teeth that the examiners did not detect. Figure 8a shows three pieces not indicated in the categorization (black boxes) but detected in the validation of the model (Figure 8b).

**Figure 9:**
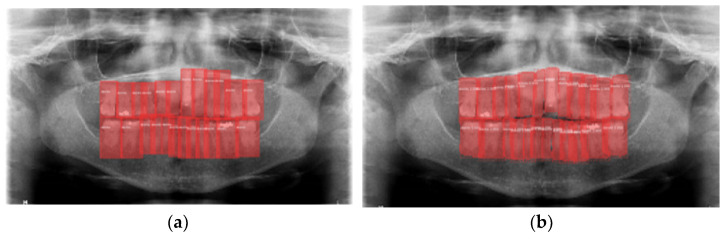
Bounding boxes: (**a**) by experts; (**b**) by the model.

Regarding the precision of bounding boxes, the model tends to demarcate more precisely than the experts who categorized the images did, as shown in Figure 9. White arrow details an example of where the model demarcates more precisely than the experts.

### 3.4. Model Execution Examples

**Figure 10:**
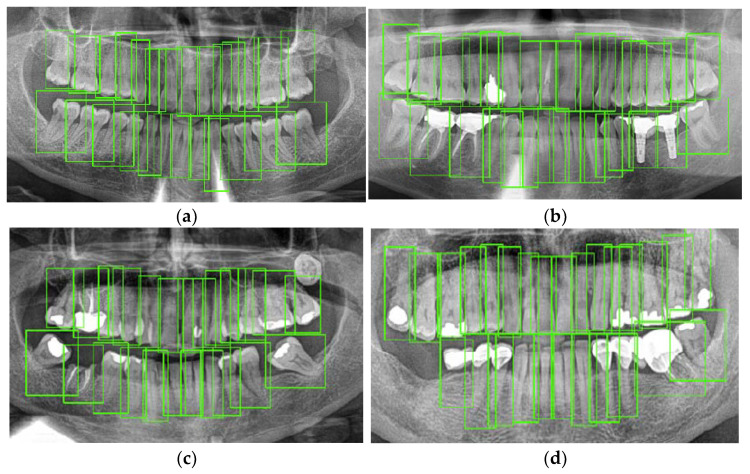
Model examples: (**a**) healthy patient; (**b**) dental implants and endodontic; (**c**) a tooth with only root; (**d**) prothesis.

Figure 10 details some examples of the results provided by the neural network employed in this study. Figure 10a corresponds to a healthy patient where all teeth are correctly detected. Figure 10b represents a patient with endodontic treatments, dental implants and a filled tooth; in this case, dental implants are detected as a presence of tooth. Figure 10c corresponds to a patient with only root in a tooth and Figure 10d corresponds to dental implants and metallic prosthesis, where the crown of the cantilever is detected as a tooth.

## 4. Discussion

The goal of this study was to build a convolutional neural network to detect the presence or absence of teeth using panoramic radiographs. A Matterport Mask RCNN and a ResNet was employed to achieve the objective of having an accuracy higher than 95%. The architecture of the model achieved an accuracy of 99.24% in teeth detection.

The first step for the use of neural networks in the field of dentistry is the correct detection of teeth, and for this reason this step must be as reliable and accurate as possible [20]. A previous literature review showed several studies that employed neural networks for teeth detection [21], which employed different type of images and neural networks.

Panoramic radiographies are very common in clinical practice due to their advantages as low radiation dose and time. This is the reason for employing this kind of image in this study besides being an image that allows visualizing the whole mouth. However, some studies employed periapical and bitewing films to detect teeth [22,23], although these kind of dental images are only used to visualize certain regions and not the entire mouth. In addition, bitewing and periapical images are commonly used to detect caries.

As with the type of image, there is also no specific number for the database used in neural network studies in dentistry. There are studies that employed 100 images to detect teeth such as Oktay, A. [24] or Muramatsu et al. [25], and other studies such as Tuzoff [3] or Chen et al. [4] that used more than 1200 images. From the best authors’ knowledge, only Zanella-Calzada et al. [26] employed an image database bigger than that used by us in this study.

It is important that the images with which the neural network learns are correctly categorized and labeled. For this, it is necessary that the training images have been previously visualized by experts, who select where the teeth appear in each image. The present study employed two examiners, with more than three years of experience in general dentistry, who independently and manually selected the presence or absence of teeth. In addition, the same criterion on teeth selection must be used by all the examiners. It is also very important to know the interexaminer agreement, that is, for the same image, how many examiners provide the same answer. Intraexaminer and interexaminer agreement is evaluated by calculating Cohen’s Kappa. According to Bulman and Osborn [27], values of Cohen’s Kappa between 0.81 and 1.00 are considered as almost perfect agreement. The present study obtained an almost perfect agreement both in the interexaminer and intraexaminer concordance.

Several studies have employed different neural network architectures to detect teeth with different type of images and obtain diverse accuracy results. According to previous reviews, the most common neural network employed to detect teeth are Mask R-CNN and faster R-CNN [21]. Jader et al. [28] employed a mask region based convolutional neural network (Mask R-CNN) to obtain the profile of each tooth employing a database of 1500 panoramic X-ray radiographies. The images used in that work were obtained from a hospital database without screening and achieved an accuracy of 0.98. Our study also employed a Mask R-CNN but combined with ResNet (Atrous Convolution). In our case, a database of 8000 panoramic radiographies, categorized by examiners was employed and an accuracy of 99.24% was achieved.

Faster R-CNN have been employed in medical applications for pulmonary nodules [29], ovarian follicles on histological images [30] or cancer [31]. In the dentistry field, authors such as Chen et al. [4], Zhang et al. [14] and Tuzoff et al. [3] worked with faster R-CNN to detect teeth, and Schwendicke et al. [32] applied Resnet to detect caries lesions.

Chen et al. [4] used faster regions with convolutional neural network features (faster R-CNN) in the TensorFlow tool package to detect and number teeth. The image database in this study was composed of 1250 periapical images obtained for treatment purposes, and an expert with more than five years of experience drew the rectangular boxes to indicate the presence of a tooth. In our case, two examiners with more than three years of experience in general dentistry selected the presence or absence of teeth. The outcome metrics in [4] were recall and precision, and obtained 0.728 and 0.771, respectively.

Zhang et al. [14] employed 1000 periapical images with faster-RCNN and Region-Based Fully Convolutional Networks (R-FCN). Although the method proposed by Zhang et al. achieved a high precision close to 95.8% and a recall of 0.961, some limitations appeared with the images with one or two teeth because the neural network did not distinguish between right and left.

Tuzoff et al. [3] employed 1352 panoramic images to detect teeth with a Faster R-CNN architecture. This study employed the same kind of images as ours. This study obtained a sensitivity of 0.9941 and a precision of 0.9945.

Miki et al. [9] employed a deep convolutional neural network (DCNN) with an AlexNet architecture for classifying tooth types on dental cone-beam computed tomography (CT) images. In that study, authors employed 42 images to train the network and 10 images to test it, and obtained a relatively high accuracy (above 80%).

Raith et al. [33] classified teeth employing a PyBrain architecture and obtained a performance of 0.93.

Muramatsu et al. [25] employed 100 dental panoramic radiographs for an object detection network using a 4-fold cross-validation method. The tooth detection sensitivity was 96.4% and the accuracy was 93.2%.

The main advantage of this study is that in the 8000 images used, the teeth have been selected as well as other variables such as root canals, fillings, etc., which will serve as inputs for subsequent studies. In addition, none of the tools available on the market were used for the categorization required in the training of the models, but rather a tool focused on the purpose of our study was created. The most important limitation of this study is that the neural network is not adapted to edentulous patients, those with temporary teeth, poor definition, with removable prostheses or with only presence of implants. Another limitation of the study is that the neural network does not detect wisdom teeth, which is proposed as a future work.

Due to the huge image database that has been categorized by the experts and employed in this first study, the authors want to adapt the architecture of the neural network to identify implants, root canals and caries, among others. Some future works are the modification of the network architecture to identify and detect implants, caries, filled teeth and root canals, and to obtain the mean age of the patient, as well as obtaining the tooth number once the errors described in relation to false positives have been solved—images that were excluded from this study.

Some false positives were obtained during the execution. Most of these false positives are due to the network detecting the presence of teeth in positions where the examiners did not select anything. These errors may be due to a lack of concentration of the examiners. These errors of lack of concentration have also been detailed and found in previous studies such as [3,14].

False positives are related to the anomalies for which the model is not trained, such as badly placed protections.

## 5. Conclusions

Based on the final accuracy achieved in this study, it is possible to conclude that the model built in this study can be used in real-life applications. The architecture used in the present study returned an almost perfect accuracy in detecting teeth on images from different devices and different pathologies and ages.

## Figures and Tables

**Table 1:** Resolution of total database image.

	Number of Images	Maximum Resolution	Minimum Resolution	Mean Resolution
DICOM 8 bit	5121	3121 × 1478	649 × 490	2699 × 1468
DICOM 12 bit	2669	304 × 2298	2105 × 1528	2682 × 1459

**Table 2:** Final parameters of the model.

Matterport Configuration Class	
Name	CoreDXnet
Backbone	Resnet101
Batch size	2
Images per GPU	2
Learning rate	0.006
Steps per epoch	200
Total epochs	60
Total steps	200
